# Olfactory Nerve—A Novel Invasion Route of *Neisseria meningitidis* to Reach the Meninges

**DOI:** 10.1371/journal.pone.0014034

**Published:** 2010-11-18

**Authors:** Hong Sjölinder, Ann-Beth Jonsson

**Affiliations:** Department of Genetics, Microbiology and Toxicology, Stockholm University, Stockholm, Sweden; Northwestern University Feinberg School of Medicine, United States of America

## Abstract

*Neisseria meningitidis* is a human-specific pathogen with capacity to cause septic shock and meningitis. It has been hypothesized that invasion of the central nervous system (CNS) is a complication of a bacteremic condition. In this study, we aimed to characterize the invasion route of *N. meningitidis* to the CNS. Using an intranasally challenged mouse disease model, we found that twenty percent of the mice developed lethal meningitis even though no bacteria could be detected in blood. Upon bacterial infection, epithelial lesions and redistribution of intracellular junction protein N-cadherin were observed at the nasal epithelial mucosa, especially at the olfactory epithelium, which is functionally and anatomically connected to the CNS. Bacteria were detected in the submucosa of the olfactory epithelium, along olfactory nerves in the cribriform plate, at the olfactory bulb and subsequently at the meninges and subarachnoid space. Furthermore, our data suggest that a threshold level of bacteremia is required for the development of meningococcal sepsis. Taken together, *N. meningitidis* is able to pass directly from nasopharynx to meninges through the olfactory nerve system. This study enhances our understanding how *N. meningitidis* invades the meninges. The nasal olfactory nerve system may be a novel target for disease prevention that can improve outcome and survival.

## Introduction

*Neisseria meningitidis* is a human-specific pathogen with the capacity to cause meningitis, which is an important cause of mortality and morbidity. Up to 50% of the survivors sustain neurological sequelae despite advances in antimicrobial therapy [1]. As a commensal bacterium, *N. meningitidis* asymptomatically colonize to the nasopharyngeal mucosa of up to 10% of the healthy population [1]. Under certain conditions, the bacteria can cross the epithelial barrier, enter and multiply in the bloodstream and disseminate to other organs including the central nervous system (CNS).

Sepsis and meningitis are the major clinical features of meningococcal disease. The high level of bacteremia together with bacterial adhesion to the endothelial cells have been suggested to be an important determinant for meningococcal traversal of the blood-brain barrier (BBB) [2], [3]. *In vitro* studies have shown that *N. meningitidis* interacts with both human vein endothelial and brain microvascular endothelial cells. Both transcellular and paracellular traversal of the host cells have been demonstrated for *N. meningitidis* [4], [5]. Pili-mediated microcolonies can recruit the cellular polarity complex upon adhesion and by this way disorganize the junction proteins at the cell-cell interface and allow bacteria to cross the BBB [4]. Moreover, bacteria have developed diverse strategies for efficient survival in the blood stream and the septic condition could induce increased BBB permeability and pleocytosis, which are the hallmarks of meningitis [6].

Apart from our knowledge of the molecular and cellular mechanisms of meningococcal traversal of the BBB, the disease pathogenesis remains incompletely understood. It is important to note that the clinical symptoms of meningococcal infection are highly diverse. More than 60% of patients develop meningitis without septic shock [7]. In cases of neonatal meningitis, meningococcal sepsis occurs in only 5–20% of patients [1]. The complex nature of the disease indicates that meningococcal invasion of the CNS can be independent of systemic bacterial dissemination in the bloodstream. An alternative non-hemetogenous traversal route to the CNS, occurring in a parallel way with or preceding systemic dissemination, could exist.

*N. meningitidis* inhabits the mucosa of the human nasopharynx, which is unique for its anatomical connection to the CNS. The posterior nasal cavity is covered by olfactory epithelium, which contains peripheral nerves of the olfactory system. The olfactory nerve axons traverse the cribriform plate, a bone behind the nose, and terminate in the olfactory bulb, which is at the inferior side of the brain. Cerebrospinal fluid (CSF), produced in the choroid plexus, acts as a cushion that protects the CNS from shocks and supports the venous sinuses. The circulation of CSF is aided by the pulsations of the choroid plexus and by the motion of the cilia of ependymal cells. The drainage of CSF from the brain plays an important role in the homeostasis and metabolism of the central nervous system. It has been assumed that CSF is absorbed from the subarachnoid compartment by arachnoid villi and granulations [8]. However, mounting evidences have proved that the lymphatic pathway external to the cranium and spinal cord has an important function in CSF absorption [9]. The association between the CSF and nasal lymph compartments in both humans and other mammalian species has been documented [10]. *In vivo* studies using animal models have shown that CSF can be removed from the cranium via transportation through the cribriform plate in association with the olfactory nerves. CSF is then absorbed into lymphatic vessels located in the sub-mucosa region of the olfactory and respiratory epithelium [11], [12].

In the present study, we aimed to investigate the dissemination route of *N. meningitidis* during invasive disease. CD46 transgenic mice that were challenged intranasally developed lethal meningitis even though no bacteria were detected in the bloodstream. We demonstrated that *N. meningitidis* is capable to invade the meninges through the olfactory nerve system. Bacterial colonization was associated with tissue damage and remodeling of N-cadherin expression patterns in the nasal olfactory epithelium.

## Results

### *N. meningitidis* induces meningitis in the absence of bacteremia

**Figure 1:**
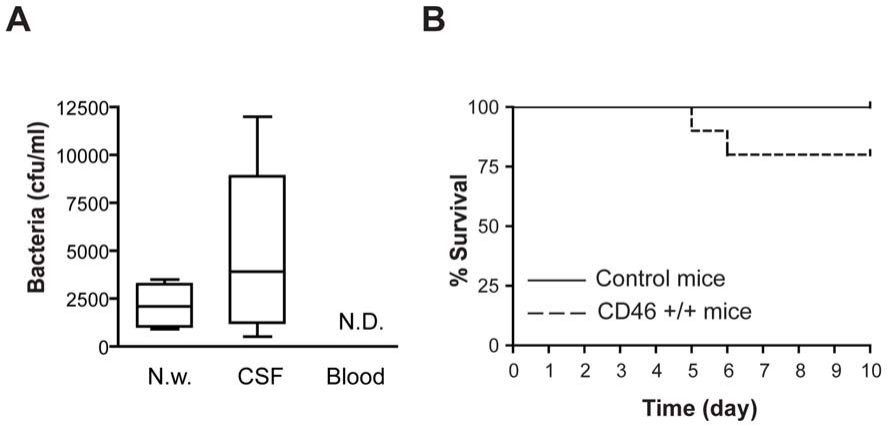
Intranasal challenge with *N. meningitidis* induces lethal disease in CD46^+/+^ mice. 10^7^ CFU of *N. meningitidis* were administrated i.n. once per day for two days to CD46 transgenic (CD46^+/+^) and non-transgenic B6C3F1 mice (Control), n = 10 per group. (A) CD46^+/+^ mice showing disease symptoms were sacrificed. Nasal washes, CSF and blood samples were collected as described in Materials and Methods. The samples were diluted and spread on GC agar plates. The number of CFU was counted the following day. Data are presented as means ± SD. The horizontal lines in the boxes represent the mean of the values. N.D., not detected. (B) Survival rates of CD46^+/+^ (bold dot line) and control (thin line) mice after i.n. challenge. Disease status was monitored for 10 days post-infection. The experiment was repeated three times with similar results.

The human cell surface receptor CD46 transgenic mouse model (CD46^+/+^) has been demonstrated to mimic many aspects of meningococcal disease in humans [13], [14], [15]. In order to study disease pathogenesis and naturally occurring infection routes, we challenged mice intranasally and monitored disease development. CD46^+/+^ and B6C3F1 mice challenged with PBS were set as uninfected control. At 3 days post-infection, nasal washes were collected from mice to determine bacterial colonization of the nasopharyngeal mucosa. Significantly higher amounts of bacteria were detected in nasal washes of CD46 transgenic mice (2680±1378 CFU/ml) compared to non-transgenic mice (432±210 CFU/ml). Further, bacteria were found in only 10% of control mice, whereas, 40% of CD46 transgenic mice were detected to carry meningococci (data not shown). We repeated the experiment and health status of all infected mice was monitored over a 10 days period. Signs of the infection, such as neck stiffness, tremble and listless, were observed in 20% of infected CD46^+/+^ mice, which had decreased survival compared to control groups, 80% versus 100% (Fig. 1B). In contrast, all B6C3F1 mice survived and no bacteria were isolated from the CSF samples (data not shown). All mice in uninfected control groups survived and no bacteria were detected in nasal washes (data not shown).

**Figure 2:**
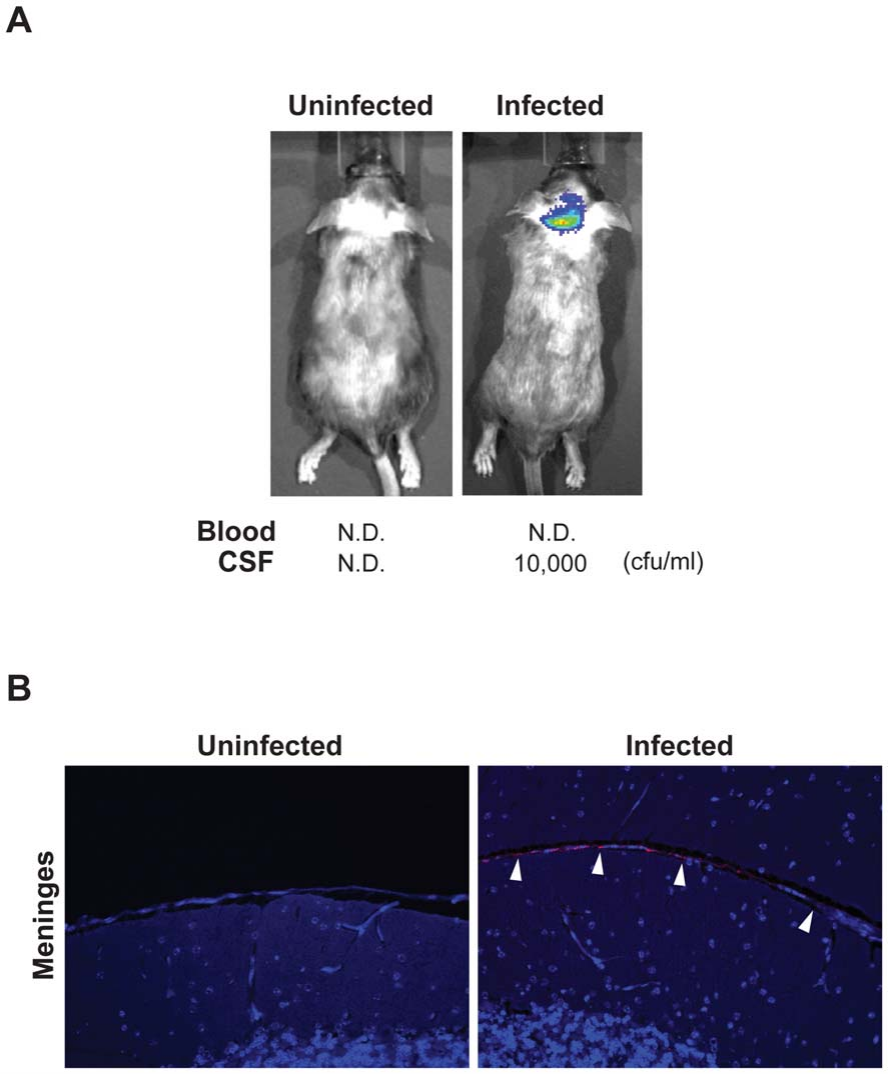
*N. meningitidis* induces meningitis in the absence of bacteremia. 10^7^ CFU of the bioluminescent FAM20 strain was given i.n. to CD46 transgenic mice (CD46^+/+^). Mice were observed using an IVIS imaging system and blood samples were take from the tail vein once per day. Mice challenged with PBS were set as uninfected control. (A) At day 4 post-infection, distinct bioluminescent signals were detected in the brain area of an infected CD46^+/+^ mouse, which had a lethal outcome. The presence of *N. meningitidis* in the CNS was confirmed by viable counts after plating serial diluted CSF sample on GC agar plates. The number of bacteria identified in the CSF and blood (CFU/ml) are presented. N.D., not detected. (B) Brain tissue was collected from mice and stained for *N. meningitidis* as described in Materials and Methods. Bacterial signals (red) were found on meningeal cells (right panel, arrowheads) of infected mice. No bacterial staining was found in the corresponding regions (left panel) of uninfected mice.

To determine the underlying pathogenesis of the disease, we measured bacterial numbers in blood of infected mice each day post infection for 10 days. Surprisingly, we were unable to detect bacteria in blood samples of all infected mice (data not shown), not even in those CD46^+/+^ mice with symptoms of lethal disease (Fig. 1A). The detection limit of the method used in our study is 500 CFU/ml of tail vein blood. Thus, we can not exclude lower (<500 CFU/ml blood) levels of bacteria in tail vein blood. Also, increased numbers of bacteria could exist in other areas of the circulatory system. Nevertheless, no bacteria were found in homogenized peripheral organs, such as liver, kidney and spleen (data not shown). However, we could detect bacteria from nasal washes and CSF of the mice that showed disease symptoms, indicating bacterial colonization of the nasopharyngeal mucosa and development of meningitis (Fig. 1A). Bacterial infection of the CNS was further confirmed by *in vivo* bioluminescent imaging. A bioluminescent FAM20 strain [15], which behaves like the wild-type strain in growth and infection behavior, was given i.n. to mice. Bioluminescence signals emitted from the bacteria were monitored *in vivo* and in real time. As shown in Fig. 2A, bacterial signals were not identified in the circulation system. In contrast, a clear bioluminescence signal was detected in the brain region. To further confirm the location of bacteria, brain tissue sections were stained with an antibody against *N. meningitidis*. Bacteria were detected in cells of the meninges (Fig. 2B, right panel, arrowheads) and in the subarachnoid space (data not shown). No bacterial signal could be detected in uninfected control mice (Fig. 2B, left panel). Thus, meningococcal dissemination to the CNS appears to be independent of bacteremia.

### A threshold level of bacteria is required for development of meningococcal septicemia

**Figure 3:**
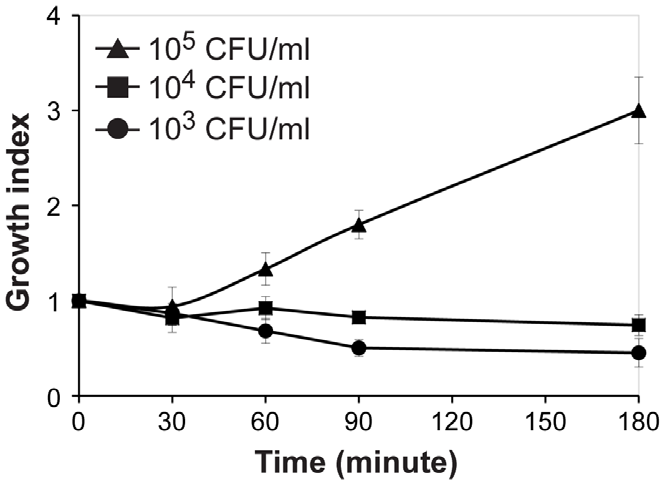
A threshold level of bacteria is required for *N. meningitidis* to grow in blood. 100 µl of *N. meningitidis* strain FAM20 suspended in cell culture medium DMEM was mixed with 100 µl of anti-coagulated whole blood collected from CD46^+/+^ mice with a final concentration of 10^3^ CFU/ml (circles), 10^4^ CFU/ml (squares) and 10^5^ CFU/ml (tri-angels), respectively. As a control, DMEM was mixed with bacteria in the same way. The samples were incubated at 37°C, 5% CO_2_ for indicated time points and the number of surviving bacteria was determined by viable count assay. Bacterial growth index at each time point was defined as (CFU after blood incubation)/(CFU after DMEM incubation).

As described above, we could not detect bacteria in the bloodstream after intranasal infection of *N. meningitidis*. A reason could be that bacteria are cleared rapidly by the host defense system in the blood and consequently cannot reach detectable levels. We therefore tested the kinetics of bacterial growth in whole blood collected from CD46 transgenic mice. At a moderate infection dose, *i. e.* 10^4^ cfu bacteria/ml, bacterial growth rate was similar in blood and in the control with cell culture medium. However, a three-fold bacterial multiplication in blood was observed after infection with 10^5^ cfu bacteria/ml. At lower infection dose, *i. e.* 10^3^ cfu/ml, bacteria could not grow in the blood (Fig. 3). Similar results were found when human blood was tested (data not shown). The impact of initial infection dose upon bacterial survival in blood was further investigated by an *in vivo* assay. Bioluminescent FAM20 bacteria were injected i.p. to CD46^+/+^ mice and bacterial growth was monitored using an *in vivo* bioluminescence imaging camera. Infection of mice with 10^6^ cfu resulted in bacterial clearance at 24 h post-infection. In contrast, when the infection dose was increased to 10^7^ cfu/mouse, around 60% of the mice developed sepsis (data not shown). These data indicate that a threshold level of bacteria is required for successful multiplication of *N. meningitidis* in blood.

### Olfactory epithelial damage upon *N. meningitidis* colonization

**Figure 4:**
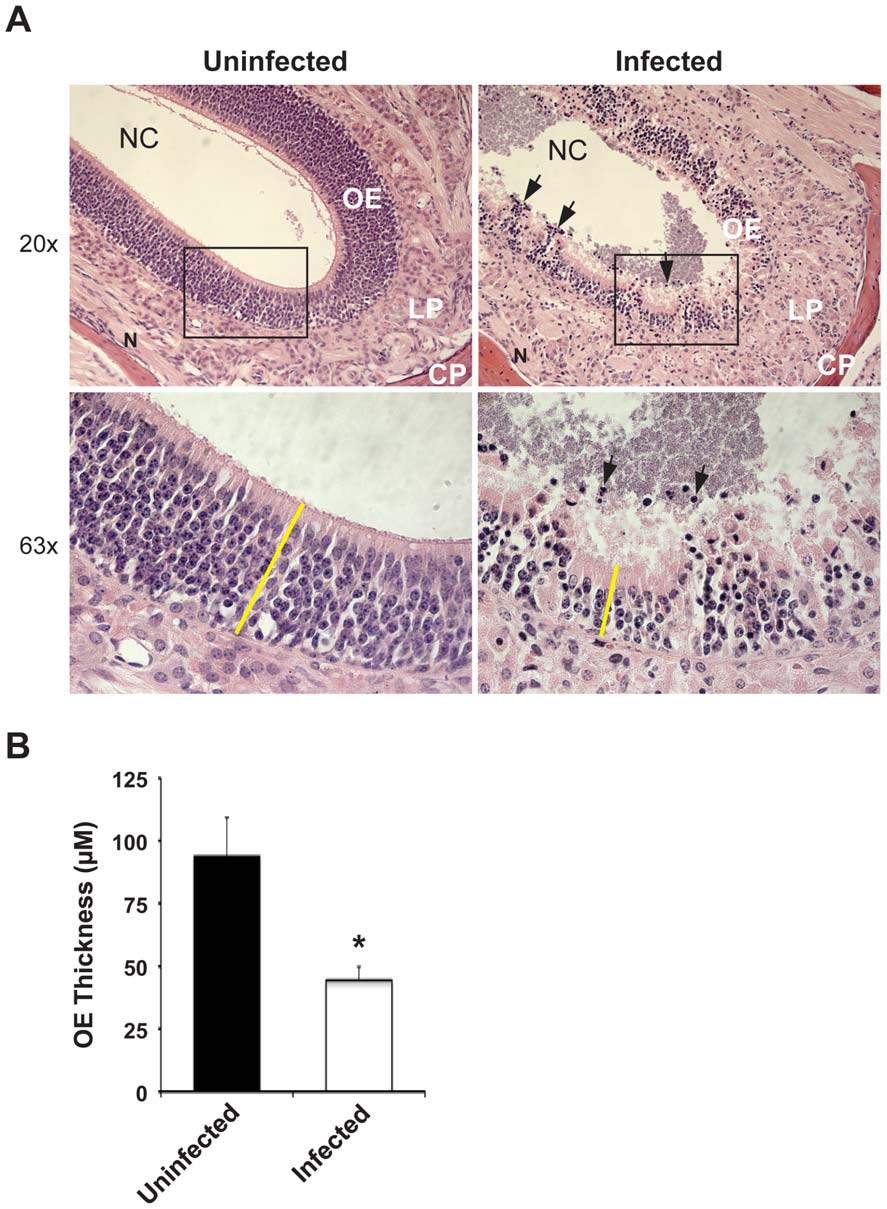
Meningococcal colonization triggers tissue damage of the olfactory epithelium. (A) 10^7^ CFU of *N. meningitidis* or PBS (uninfected control) was administrated i.n. to CD46 transgenic mice. At day 3 post-challenge, nasal tissue sections were collected and stained with Hematoxylin and Eosin. Hematoxylin stains negatively charged nuclei dark blue and eosin stains other tissue structures pink. The olfactory epithelium is thereby visualized as a region with a high density of heavily hematoxylin-stained cells. Images show the olfactory epithelial (OE) region of nasal mucosa and the luminal space of the nasal cavity (NC). Epithelial damage and atrophy was induced upon bacterial infection (right panels). Higher magnification (63×) of the box in upper panels is shown in lower panels. Arrows indicate infiltrated cells. Yellow bar: thickness of the OE. Abbreviations: LP, lamina propria; CP, cribriform plate; N, olfactory nerve. (B) Thickness of the olfactory epithelium was measured using a Carl Zeiss Axio Vision 2.05 image analysis system (Zeiss). Upon meningococcal infection, the thickness of the OE is significantly decreased compared to the uninfected mice (A, left panels). *, *p*<0.05, nonparametric Mann-Whitney test.

Nasopharyngeal mucosa is the natural niche for *N. meningitidis*. More than 50% of the nasal cavity surface in mice is covered by olfactory epithelium [16], which is a unique part of the nervous system containing olfactory sensory neurons. Bacterial colonization and histopathological alterations in sections of head tissue were therefore studied. Mice infected with meningococci had conspicuous morphological changes and tissue lesions in the olfactory epithelial region. Polymorphonuclear cells were abundant in the olfactory epithelium and in the nasal cavity resulting in an atypical multilayer structure (Fig. 4A, right panels). These characters were not observed in the saline vehicle-treated control mice (Fig. 4A, left panels). Furthermore, epithelial atrophy was observed upon infection, the average thickness of the olfactory epithelium after infection was reduced by more than 50% compared with control mice (Fig. 4B).

### *N. meningitidis* invades the meninges through the olfactory nerves

**Figure 5:**
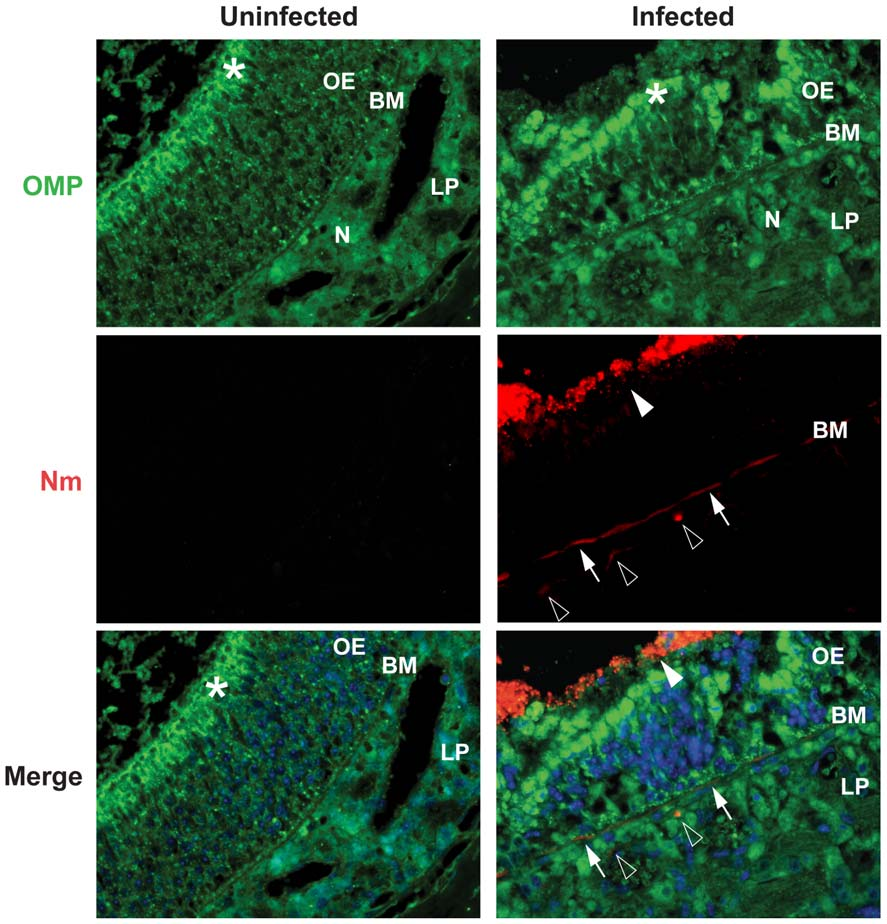
Colonization and invasion of *N. meningitidis* to the olfactory mucosa. 10^7^ CFU of *N. meningitidis* were given i.n. to CD46 transgenic mice (Infected). At day 3 post-challenge, nasal tissue sections were collected and stained for *N. meningitidis* as described in Materials and Methods. Olfactory epithelium (OE) was determined by immunofluorescence staining using an antibody against olfactory marker protein (OMP), which recognizes mature cells of the olfactory sensory neuron and their dendrites in the olfactory epithelium. Strong continuous OMP immunoreactivity (green) could be seen in the apical olfactory epithelium (OE, *) and bundles of olfactory nerves (N) in lamina propria (LP). Nuclei were stained with DAPI. Bacterial signals (Nm, red) were detected on the surface of the olfactory epithelium (arrwohead), at the basement layer (BM, arrows) and in lamina propria (LP, empty triangles). No bacterial staining was found in the corresponding regions of uninfected mice.

**Figure 6:**
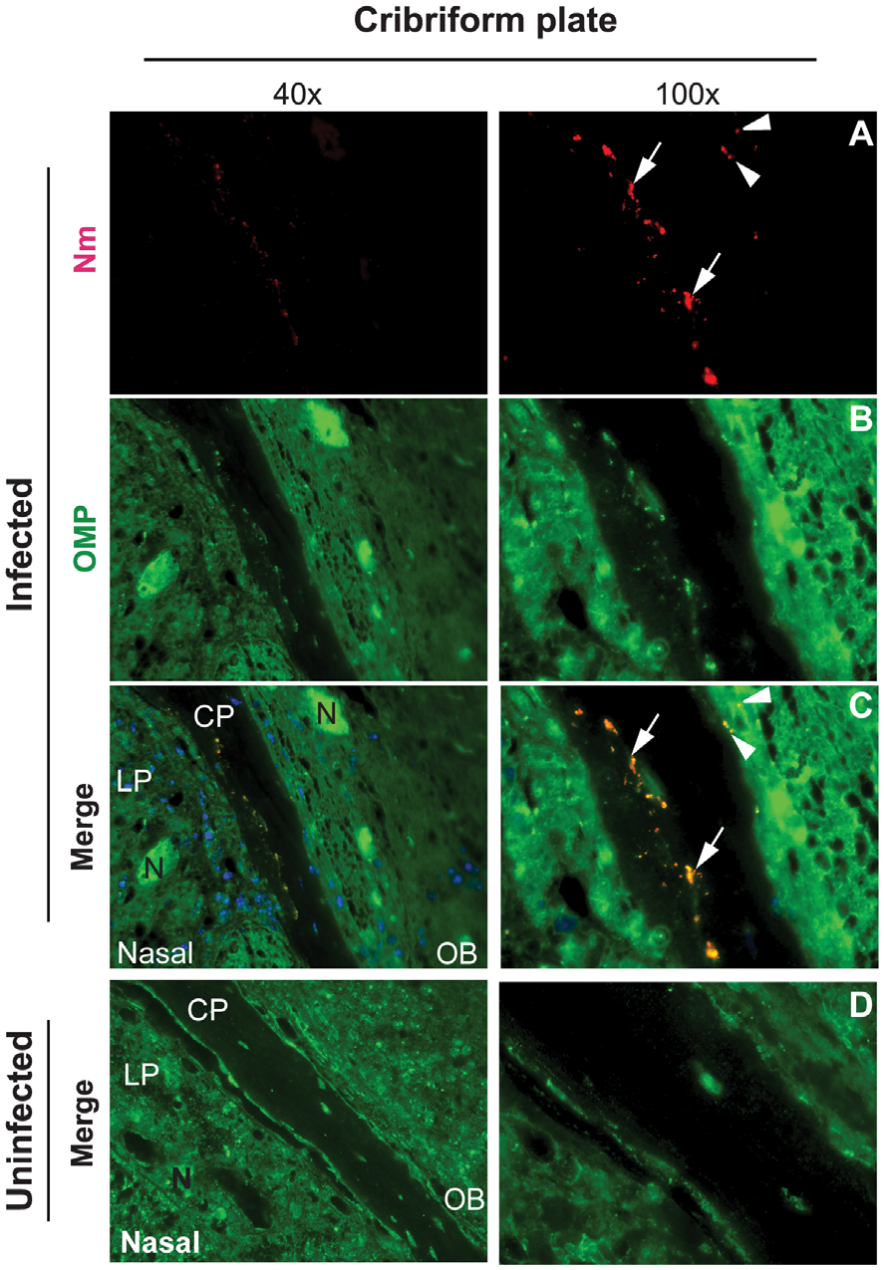
*N. meningitidis* invades the CNS through the olfactory nerve. CD46 transgenic mice were challenged i.n. with 10^7^ CFU of *N. meningitidis* (Infected). At three days post-infection, the head tissue samples were prepared by sectioning through the midsagittal plane to give a complete anatomic structure between the nasopharyngeal region (nasal) and the olfactory bulb (OB). (A) Bacteria (Nm, red signal) were detected in the cribriform plate (arrows) and at the edge of olfactory bulb (arrowheads). (B) Olfactory nerve system was determined by using an antibody against olfactory marker protein (OMP, green signal). (C) In merged images, all bacterial signals co-localized with OMP, a marker for the olfactory nerve. (D) No bacterial staining was found in the corresponding regions of uninfected mouse. Abbreviations: LP, lamina propria; CP, cribriform plate; OB, olfactory bulb; N, olfactory nerve.

As shown above, although bacteria reached the CNS, they could not be detected in blood. Furthermore, tissue damage of the olfactory epithelium was observed upon meningococcal infection. We postulated that the olfactory nervous system, a structural and functional connection between the nasopharynx and the CNS could be a pathway for bacterial dissemination. Three days after infection, mice were sacrificed and the head tissue was split at the midsagittal plane, giving a complete anatomic structure between nasopharyngeal and the CNS. By immunohistochemistry straining of the tissue sections, *N. meningitidis* was detected at the olfactory epithelium of the nasal cavity (Fig. 5, red signal, arrowhead). Moreover, bacterial signals were also found at the basement membrane (BM) (Fig. 5, arrows) and in lamina propria (LP) (Fig. 5, empty triangles). We could not detect obvious bacterial signals in the olfactory epithelium (OE) (Fig. 5). We used an antibody recognizing olfactory marker protein (OMP) to stain the olfactory nerve system, including olfactory receptor cells in the olfactory epithelium, bundles of axons in lamina propria, the cribriform plate and the olfactory bulb. The olfactory receptor cells are bipolar neurons that form bundled axons at the base of the cells, penetrate the cribriform plate and reach the olfactory bulb of the brain. The above region was therefore studied carefully. Strong bacterial signals (red) were identified in the cribriform plate and at the nerve fiber layer of the olfactory bulb (Fig. 6A). All observed bacterial signals co-localized with the staining of olfactory marker protein, indicating that bacteria disseminated to the CNS through the olfactory nerve (Fig. 6B and C). We did not observe obvious bacterial signals in olfactory bulb tissue (Fig. 6A and C). In control mice, no bacterial signal was detected (Fig. 6D).

### Decreased expression of N-cadherin in the olfactory epithelium upon meningococcal infection

**Figure 7:**
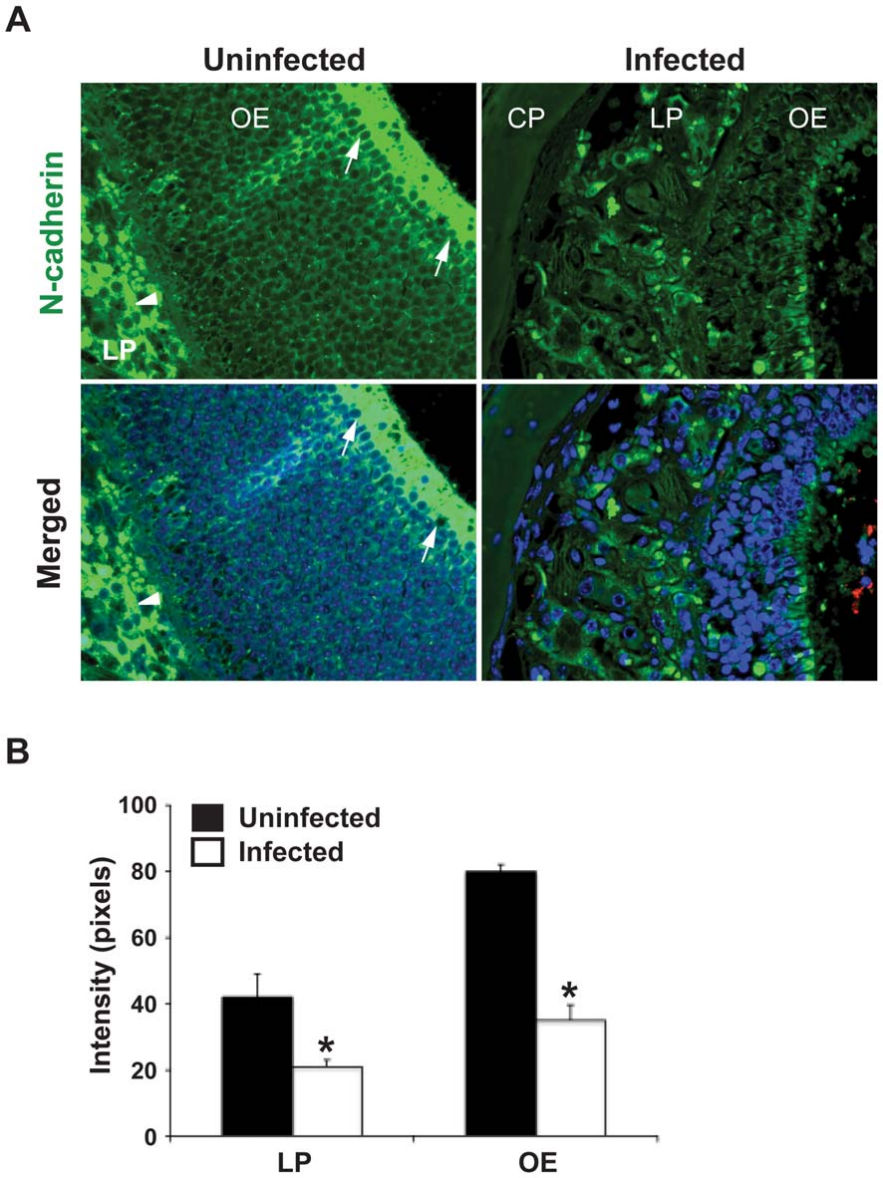
Depletion of N-cadherin in olfactory epithelium upon *N. meningitidis* infection. CD46 transgenic mice were challenged i.n. with 10^7^ CFU of *N. meningitidis*. Mice challenged with PBS were set as uninfected control. At day 3 post-challenge, head tissue was collected and tissue sections were stained using N-cadherin and *N. meningitidis* antibodies as described in Materials and Methods. (A) In uninfected mice (left panels), strong and continuous N-cadherin staining (green) was identified in the apical layer (arrows) of the olfactory epithelium (OE) and bundles of olfactory nerve (arrowheads) in lamina propria (LP). In infected mice (right panels), the N-cadherin signal was significantly decreased in the corresponding regions. In merged image, bacteria (red) were detected at the olfactory mucosa surface of infected mice. Nuclei were stained with DAPI. (B) Quantification of N-cadherin staining intensity by software Image J. After infection, expression of N-cadherin was significantly decreased in both OE and the LP region. The data are presented as mean ± SD. Significant reductions of signal intensity compared with uninfected mice are indicated with asterisks (*, *p*<0.05, nonparametric Mann-Whitney test). Abbreviations: OE, olfactory epithelium; LP, lamina propria; CP, cribriform plate.

By using human brain vascular endothelial cells, Coureuil *et al*. [4] showed that pilus-mediated adhesion of *N. meningitidis* can lead to the formation of ectopic intercellular junction domains at the site of bacteria-cell interaction. This resulted in a depletion of junction proteins at the cell-cell interface, and an opening of the intercellular junctions, which would enable bacteria to cross the BBB and invade the meninges through paracellular gaps. To extend this finding, we next investigated whether a similar molecular mechanism of bacterial invasion exists under a non-hematogenous pathway. The expression of junction proteins (N-cadherin, ZO-1 and β-catenin) in the olfactory epithelium was analyzed by immunofluorescence staining. In uninfected mice, N-cadherin was expressed mainly in apical olfactory epithelium and bundles of olfactory nerve in lamina propria (Fig. 7A, left panels). After meningococcal infection, expression of N-cadherin was dramatically affected (Fig. 7A, right panels). Signal intensity of N-cadherin in the olfactory epithelium (OE) and the lamina propria (LP) was significantly reduced upon meningococcal infection compared to the uninfected control (Fig. 7B). The expression levels of ZO-1 and β-catenin in olfactory epithelium were not obviously affected upon infection (data not shown) indicating that the down-regulation of N-cadherin is infection specific.

## Discussion

Given the complex nature of the meningococcal disease, it has long been hypothesized that *N. meningitidis* could enter into the CNS via a non-haemetogenous invasion pathway [17]. By using a humanized mouse disease model, we show here that *N. meningitidis* can invade the meninges through the olfactory nerve system. Induction of meningitis was confirmed by bacterial viable count assay of CSF samples, *in vivo* bioluminescence imaging and immunofluorescence staining of the brain tissue. An alternative explanation for our findings is that bacterial colonization of the olfactory nerve occurred as a second event following CNS penetration by other pathways. We find this highly unlikely since we could not detect bacteria in blood or peripheral organs. Upon bacterial infection, tissue lesions and remodeling of the intracellular junction protein N-cadherin was observed in nasal mucosa, especially at the olfactory epithelium, which functionally and anatomically connects the CNS and nasopharynx. Structural damages could trigger bacterial entry into the CNS. Indeed, bacteria were detected not only at the surface of olfactory epithelial cells, but also in the sub-mucosa region. Bacteria were further identified along the olfactory nerves in the cribriform plate, at the peripheries of the olfactory bulb and at the meninges and subarachnoid space. Our results highlight the important role of olfactory nervous system in meningococcal infection and might explain the presence of meningeal infection in patients with negative blood cultures.

Olfactory nerves together with the lymphatic system in nasal submucosa build up an anatomical and functional connection between respiratory epithelium and the CNS. Many neurotropic pathogens make use of this physiological route to invade the CNS. In line with this study, Marra [18] demonstrated that *Streptococcus pneumoniae* can spread to the CNS and cause meningitis without a bacteremic state. Furthermore, it has been suggested that the interaction of pneumococci with neuronal gangliosides is essential for direct bacterial entry into the CNS along olfactory nerves [19]. Certain viruses, such as herpes simplex virus and influenza A virus, are able to reach the brain through the olfactory epithelium, along the nerve fibers of the olfactory, vagal and trigeminal [20], [21], [22]. In a murine disease model, *Naegleria fowleri* can invade the CNS through olfactory nerves during the early stages of primary amebic meningoencephalitis (PAM). The amebas could be observed in the submucosal nerve plexus, olfactory nerves within the cribriform plate, and the olfactory bulb as early as 24 h post-infection [23].

The CNS is covered by meninges and is highly protected from most microorganisms and toxins that are circulating in the bloodstream because of the protective properties of the blood-brain barrier (BBB), which is a structural and functional barrier formed by the microvascular endothelium, astrocytes and pericytes. Intercellular tight junctions together with a low rate of pinocytosis make the BBB impermeable for large and hydrophilic molecules as well as for most pathogens [24], [25]. Adhesion of meningococci to human brain vascular endothelial cells was shown to cause remodeling of cell-cell junction proteins and subsequent formation of gaps between infected cells, allowing bacteria to cross the BBB [4]. In line with the *in vitro* studies, we found that bacterial colonization plays an important role in the development of meningitis *in vivo*, since invasive disease was only observed in colonized mice. Moreover, expression levels of N-cadherin in the olfactory mucosa were attenuated upon meningococcal infection. Meningococcal colonization led to conspicuous epithelial damage and tissue atrophy especially at the olfactory epithelial region. These findings are in accordance with a recently published study by Schubert-Unkmeir *et al*. [26], who revealed that cellular inflammatory mediators, for example matrix metalloproteinases, degrade intercellular junction proteins. This results in endothelial cell disruption and hence facilitates bacterial invasion. It was therefore speculated that infection-induced tissue damage together with remodeling of the host cell cytoskeleton and junction proteins could be a general mechanism used by *N. meningitidis* during tissue invasion.

Membrane cofactor protein CD46 is used as a cell receptor by a number of important pathogenic bacteria and viruses, including *N. meningitidis* [27]. In this study, meningococci bound to the olfactory mucosa even though CD46 is expressed on the basolateral surface of the epithelium [13]. Our data indicates that CD46 might not be involved in mediating the initial bacterial adhesion. Indeed, other cell receptors or strategies have been shown to participate in bacteria-host cell interaction. Carcinoembryonic antigen-related cell adhesion molecules (CEACAMs) can function as a receptor to mediate intimate binding of meningococci to epithelial cells through Opa proteins [28]. Type IV pili component PilV can trigger cell membrane reorganization and by this way enhances bacterial cohesion [2]. It might be hypothesized that the observed epithelial damage and decreased expression of junction protein allows for bacterial binding to CD46. How these events facilitate bacterial invasion will need further investigation. CD46 has also been considered as a regulator of the complement system by facilitating factor I-mediated inactivation of the activated complement proteins C3b and C4b [29]. Many pathogens might recruit extracellular CD46 as a strategy to survive host complement defense as it has been demonstrated that both *N. meningitidis* and *S. pyogenes* can induce systemic infection in CD46 transgenic mice [15], [30], [31].

Several non-CD46 expressing mice models have been evaluated to study the earlier stage of meningococcal infection by an intranasal infection route. Infant NIH mice developed both sepsis and meningitis, however, the requirement for lung colonization to precede bacteremia limit the use of this model to study meningococcal pathogenesis. Furthermore, the impact of the premature immune system to the disease development is not clear [32]. When adult Swiss Webster mice were studied, even though a long-term mucosal colonization was maintained, no invasive disease could be observed [33]. Invasive disease can be observed in adult C57BL and Swiss Webster mice following intraperitoneal infection, however, enhancement substances with unknown effects on the host immune response are essential [34]. We have shown that CD46 transgenic mice are susceptible to meningococcal infection. Comparable disease presentations, such as sepsis, meningitis and upper respiratory mucosal colonization, have been observed in adult mice after i.p. or i.v. challenge without using any enhancement substances [13], [15]. In this study we showed the adult CD46 transgenic mice can also develop invasive meningococcal disease following intranasal infection. Meningitis can be induced even though no bacteria in the blood or peripheral organs can be detected. It is likely that a threshold level of bacteremia is required for the development of sepsis as demonstrated in this study by both *in vivo* and *in vitro* assays. Strain-specific capacity to survive in the blood stream could be another reason since JB515, a serogroup W-135 strain used in the previous work, did result in bacteremia [13]. Nevertheless, species-specific differences of the complement system between mouse and human should be considered when conclusions are drawn from animal models.

Taken together, the present study provides compelling evidence that *N. meningitidis* is able to invade the CNS and induce meningitis through the olfactory nerve system in the absence of bloodstream dissemination. Further studies of the bacterial and cellular components involved in this novel invasion route could lead not only to a complete understanding of the pathophysiology of the disease, but also more efficient therapeutic strategies.

## Materials and Methods

### Ethics Statement

Mice experiments described in the present study were conducted at the animal facility of Wenner-Grens Institute, Stockholm University. Animal care and experiments were conducted adhering to the institution's guidelines for animal husbandry. All protocols were approved by the Swedish Ethical Committee on Animal Experiments (Approval ID: N380/08).

### Bacterial strains and growth conditions

*N. meningitidis* FAM20 belongs to serogroup C, MLST sequence type 11. The bioluminescent FAM20 strain, FAM20::Lux, has been described previously [15] and behaves like the wild-type strain in growth and infection behavior. Bacteria were grown at 37°C in a 5% CO2 atmosphere on GC agar (Difco) supplemented with Kelloggs [35].

### Mouse strains

The hCD46Ge transgenic mouse line (CD46^+/+^) was created using B6C3F1 hybrids. It harbors the complete human CD46 gene and expresses CD46 in a human-like pattern [36]. The F1 generation of C57BL/6 and C3H/Hen mice, *i.e.* B6C3F1, was used as non-transgenic control in this study.

### Mouse infection studies

*N. meningitidis* grown on GC agar was suspended in phosphate-buffered saline (PBS). Each mouse was challenged intranasally (i.n.) with 10^7^ bacteria in 10 µl PBS once per day for two days. Prior to challenge, mice were treated with antibiotics as previously described [13]. The health status of the mice was closely monitored for 10 days and mice were sacrificed as soon as the end point was reached. CD46^+/+^ and B6C3F1 mice challenged with PBS alone were set as uninfected control. Experiments were performed with 6–8 week old mice (n = 10–12 mice per group) and repeated three times.

### Detection of meningococci in blood

After bacterial challenge, 5 µl of blood sample was collected from the tail vein each day during the whole experiment. The blood was diluted in 245 µl of GC liquid, and 100 µl was plated on a GC agar plate. The remaining sample was used for further plating of serial dilutions. Plates were incubated over night at 37°C in a 5% CO_2_ atmosphere. The number of colony forming units (CFU) was counted next day. The limit of detection is 500 CFU/ml blood.

### Detection of meningococci in cerebrospinal fluid

Cerebrospinal fluid were collected either at the end of the experiment or at the point when the disease signs were observed. Mice were sacrificed by inhalation of isoflurane (Forene, Abbott) and the skin of the back neck was disinfected with ethanol. The skull was exposed through a midline incision and the cisterna magna was punctured and approximately 1 to 2 µl of CSF was collected from each mouse. The CSF samples, which were immediately suspended in 100 µl of GC liquid, were examined for the absence of red blood cells before spreading on the GC agar plates. The number of bacterial CFU was determined after an overnight incubation at 37°C in a 5% CO_2_ atmosphere.

### Detection of meningococci in nasal washes

Nasal washes were collected either at the end of the experiment or at the point when the disease signs were observed. Mice were sacrificed by inhalation of isoflurane and the skin of the front neck was disinfected with ethanol. The trachea of mouse was then isolated, 200 µl of PBS was rinsed from the lower end of the trachea and collected from the external narines. The nasal washes were immediately checked for the absence of red blood cells and spread on the GC agar plates containing kanamycin (50 µg/ml) in order to inhibit the overgrow of the normal flora. The number of bacterial CFU was determined after an overnight incubation at 37°C in a 5% CO_2_ atmosphere.

### Bioluminescence imaging

Mice were anaesthetized with isoflurane and imaged for a maximum of 2 min using an IVIS imaging system (Xenogen Corporation/Caliper Life Sciences) according to the manufacturer's instructions. The dark fur on the back and the head of the mice was shaved away in order to increase the sensitivity of signal detection.

### Histology, light microscopy and immunofluorescence staining

Mice were sacrificed and perfused using 4% formaldehyde in PBS. The head was split in the midsagittal plane, fixed in 4% paraformaldehyde, decalcified in 10% fomic acid and embedded in paraffin. Sequential tissue sections obtained from three mice were analyzed and images from one mouse are presented. For light microscopy, tissue sections were stained with hematoxylin and eosin. Hematoxylin stains negatively charged nuclei dark blue and eosin stains other tissue structures pink. The olfactory epithelium is thereby visualized as a region with a high density of heavily hematoxylin-stained cells. In order to investigate epithelial atrophy, six fields of epithelial layer without obvious neutrophils influx, which could affects the epithelial integrity, were randomly selected and the thickness of the epithelium was analysed by a Carl Zeiss Axio Vision 2.05 image processing and analysis system (Zeiss). The average thickness of the olfactory epithelium is presented.

The primary antibodies applied in immunofluorescence staining are: Goat anti-Olfactory Marker Protein (1∶100, Waco Chemicals, USA); Mouse anti-N-cadherin (1∶50, Sigma-Aldrich); Goat anti-ZO-1 (1∶100, Santa Cruz Biotechnology); Goat anti-β-catenin (1∶100, Santa Cruz Biotechnology) and Rabbit anti-*N. meningitidis* (1∶100, USBiological). Before staining, tissue sections were blocked with Image-iT™ FX Signal Enhancer (Invitrogen) for 30 min at room temperature (RT), and subsequently incubated with primary antibody diluted in PBS for 1 h at RT. After three washes in PBS for 5 min and staining with Alexa Fluor 488-, 594-conjugated secondary antibody (1∶100 diluted in PBS, Invitrogen) for 1 h at RT, slides were washed and mounted in DAPI containing Prolong gold anti-fade reagent (Invitrogen). Controls for unspecific binding by both primary and secondary antibodies were included in each staining. Image acquisition was performed by a Carl Zeiss Axio Vision 2.05 image processing and analysis system (Zeiss).

### Growth kinetics of *N. meningitidis* in whole blood

Whole blood was obtained from healthy volunteers without previous immunization with anti-meningococcal vaccine or collected from the orbital vein of the mice. Heparin (10 units/ml blood) was added to prevent coagulation. *N. meningitidis* suspensions were prepared in DMEM cell culture medium (Invitrogen), and mixed with the whole blood at a ratio of 1∶1 to final concentrations of 10^3^ to 10^5^ bacteria/ml blood. The bacterial solution was then incubated at 37°C in a 5% CO_2_ atmosphere. At different time points, bacteria were serially diluted in GC liquid and spread on GC agar plates to enumerate the surviving colonies after an overnight incubation.

### Statistical analysis

The nonparametric Mann-Whitney test was used to analyze differences between groups. All tests were two-tailed and data were considered significant if *P* value was less than 0.05.

## References

[1] Rosenstein NE, Perkins BA, Stephens DS, Popovic T, Hughes JM (2001). Meningococcal disease.. N Engl J Med.

[2] Mikaty G, Soyer M, Mairey E, Henry N, Dyer D (2009). Extracellular bacterial pathogen induces host cell surface reorganization to resist shear stress.. PLoS Pathog.

[3] van Deuren M, Brandtzaeg P, van der Meer JW (2000). Update on meningococcal disease with emphasis on pathogenesis and clinical management.. Clin Microbiol Rev.

[4] Coureuil M, Mikaty G, Miller F, Lecuyer H, Bernard C (2009). Meningococcal type IV pili recruit the polarity complex to cross the brain endothelium.. Science.

[5] Nikulin J, Panzner U, Frosch M, Schubert-Unkmeir A (2006). Intracellular survival and replication of Neisseria meningitidis in human brain microvascular endothelial cells.. Int J Med Microbiol.

[6] Nishioku T, Dohgu S, Takata F, Eto T, Ishikawa N (2009). Detachment of brain pericytes from the basal lamina is involved in disruption of the blood-brain barrier caused by lipopolysaccharide-induced sepsis in mice.. Cell Mol Neurobiol.

[7] Stephens DS, Greenwood B, Brandtzaeg P (2007). Epidemic meningitis, meningococcaemia, and Neisseria meningitidis.. Lancet.

[8] Tubbs RS, Hansasuta A, Stetler W, Kelly DR, Blevins D (2007). Human spinal arachnoid villi revisited: immunohistological study and review of the literature.. J Neurosurg Spine.

[9] Johnston M, Papaiconomou C (2002). Cerebrospinal fluid transport: a lymphatic perspective.. News Physiol Sci.

[10] Johnston M, Zakharov A, Papaiconomou C, Salmasi G, Armstrong D (2004). Evidence of connections between cerebrospinal fluid and nasal lymphatic vessels in humans, non-human primates and other mammalian species.. Cerebrospinal Fluid Res.

[11] Nagra G, Koh L, Zakharov A, Armstrong D, Johnston M (2006). Quantification of cerebrospinal fluid transport across the cribriform plate into lymphatics in rats.. Am J Physiol Regul Integr Comp Physiol.

[12] Zakharov A, Papaiconomou C, Johnston M (2004). Lymphatic vessels gain access to cerebrospinal fluid through unique association with olfactory nerves.. Lymphat Res Biol.

[13] Johansson L, Rytkonen A, Bergman P, Albiger B, Kallstrom H (2003). CD46 in meningococcal disease.. Science.

[14] Johansson L, Rytkonen A, Wan H, Bergman P, Plant L (2005). Human-like immune responses in CD46 transgenic mice.. J Immunol.

[15] Sjolinder H, Jonsson AB (2007). Imaging of disease dynamics during meningococcal sepsis.. PLoS One.

[16] Adams DR (1972). Olfactory and non-olfactory epithelia in the nasal cavity of the mouse, Peromyscus.. Am J Anat.

[17] Filippidis A, Fountas KN (2009). Nasal lymphatics as a novel invasion and dissemination route of bacterial meningitis.. Med Hypotheses.

[18] Marra A, Brigham D (2001). Streptococcus pneumoniae causes experimental meningitis following intranasal and otitis media infections via a nonhematogenous route.. Infect Immun.

[19] van Ginkel FW, McGhee JR, Watt JM, Campos-Torres A, Parish LA (2003). Pneumococcal carriage results in ganglioside-mediated olfactory tissue infection.. Proc Natl Acad Sci U S A.

[20] Iwasaki T, Itamura S, Nishimura H, Sato Y, Tashiro M (2004). Productive infection in the murine central nervous system with avian influenza virus A (H5N1) after intranasal inoculation.. Acta Neuropathol.

[21] Mori I, Nishiyama Y, Yokochi T, Kimura Y (2005). Olfactory transmission of neurotropic viruses.. J Neurovirol.

[22] Park CH, Ishinaka M, Takada A, Kida H, Kimura T (2002). The invasion routes of neurovirulent A/Hong Kong/483/97 (H5N1) influenza virus into the central nervous system after respiratory infection in mice.. Arch Virol.

[23] Jarolim KL, McCosh JK, Howard MJ, John DT (2000). A light microscopy study of the migration of Naegleria fowleri from the nasal submucosa to the central nervous system during the early stage of primary amebic meningoencephalitis in mice.. J Parasitol.

[24] Nassif X, Bourdoulous S, Eugene E, Couraud PO (2002). How do extracellular pathogens cross the blood-brain barrier?. Trends Microbiol.

[25] Rubin LL, Staddon JM (1999). The cell biology of the blood-brain barrier.. Annu Rev Neurosci.

[26] Schubert-Unkmeir A, Konrad C, Slanina H, Czapek F, Hebling S (2010). Neisseria meningitidis induces brain microvascular endothelial cell detachment from the matrix and cleavage of occludin: a role for MMP-8.. PLoS Pathog.

[27] Kallstrom H, Liszewski MK, Atkinson JP, Jonsson AB (1997). Membrane cofactor protein (MCP or CD46) is a cellular pilus receptor for pathogenic Neisseria.. Mol Microbiol.

[28] Muenzner P, Dehio C, Fujiwara T, Achtman M, Meyer TF (2000). Carcinoembryonic antigen family receptor specificity of Neisseria meningitidis Opa variants influences adherence to and invasion of proinflammatory cytokine-activated endothelial cells.. Infect Immun.

[29] Liszewski MK, Leung M, Cui W, Subramanian VB, Parkinson J (2000). Dissecting sites important for complement regulatory activity in membrane cofactor protein (MCP; CD46).. J Biol Chem.

[30] Johnson JB, Grant K, Parks GD (2009). The paramyxoviruses simian virus 5 and mumps virus recruit host cell CD46 to evade complement-mediated neutralization.. J Virol.

[31] Lovkvist L, Sjolinder H, Wehelie R, Aro H, Norrby-Teglund A (2008). CD46 Contributes to the severity of group A streptococcal infection.. Infect Immun.

[32] Mackinnon FG, Gorringe AR, Funnell SG, Robinson A (1992). Intranasal infection of infant mice with Neisseria meningitidis.. Microb Pathog.

[33] Yi K, Stephens DS, Stojiljkovic I (2003). Development and evaluation of an improved mouse model of meningococcal colonization.. Infect Immun.

[34] Brodeur BR, Larose Y, Tsang P, Hamel J, Ashton F (1985). Protection against infection with Neisseria meningitidis group B serotype 2b by passive immunization with serotype-specific monoclonal antibody.. Infect Immun.

[35] Kellogg DS, Cohen IR, Norins LC, Schroeter AL, Reising G (1968). Neisseria gonorrhoeae. II. Colonial variation and pathogenicity during 35 months in vitro.. J Bacteriol.

[36] Mrkic B, Pavlovic J, Rulicke T, Volpe P, Buchholz CJ (1998). Measles virus spread and pathogenesis in genetically modified mice.. J Virol.

